# The Role of microRNAs as Potential Biomarkers in Diffuse Large B-Cell Lymphoma

**DOI:** 10.3390/ncrna12010002

**Published:** 2026-01-07

**Authors:** Eirini Panteli, Epameinondas Koumpis, Vasileios Georgoulis, Georgios Petros Barakos, Evangelos Kolettas, Panagiotis Kanavaros, Alexandra Papoudou-Bai, Eleftheria Hatzimichael

**Affiliations:** 1 Department of Hematology, Faculty of Medicine, School of Health Sciences, University of Ioannina, 45500 Ioannina, Greece; eir.panteli@gmail.com (E.P.); an.koumpis@uoi.gr (E.K.); vasileios.georgoulis@gmail.com (V.G.); 2 First Department of Internal Medicine, General Hospital of Piraeus “Tzaneio”, 18536 Piraeus, Greece; bargeo46@gmail.com; 3 Laboratory of Biology, Faculty of Medicine, School of Health Sciences, Institute of Biosciences, University Centre for Research and Innovation, University of Ioannina, 45110 Ioannina, Greece; ekoletas@uoi.gr; 4 Biomedical Research Institute, Foundation for Research and Technology, 45110 Ioannina, Greece; 5 Department of Anatomy-Histology-Embryology, Faculty of Medicine, School of Health Sciences, University of Ioannina, 45110 Ioannina, Greece; pkanavar@uoi.gr; 6 German Medical Institute, 1, Nikis Avenue, 4108 Limassol, Cyprus; apapoudoubai@gmail.com; 7 Computational Medicine Center, Sidney Kimmel Medical College, Thomas Jefferson University, Philadelphia, PA 19107, USA

**Keywords:** miRNAs, DLBCL, lymphoma, miR-155, miR-21, miR-17-92, non-Hodgkin, non-coding RNA, miR-34

## Abstract

Diffuse large B-cell lymphoma (DLBCL) is the most common and clinically aggressive subtype of non-Hodgkin lymphoma (NHL). While novel therapies such as rituximab and polatuzumab vedotin have led to improved outcomes, approximately 35% of patients eventually develop relapsed or refractory disease. MicroRNAs (miRNAs), a class of endogenous single-stranded RNAs approximately 22 nucleotides in length, play a pivotal role in the regulation of gene expression at the post-transcriptional level through interactions with complementary target RNAs and contribute significantly to the development, progression, and treatment response of DLBCL. Oncogenic miRNAs, such as miR-155, miR-21, and the miR-17–92 cluster, promote proliferation, survival, immune evasion, and therapy resistance by modulating pathways including PI3K/AKT, NF-κB, and MYC. Conversely, tumor-suppressive miRNAs such as miR-34a, miR-144, miR-181a, and miR-124-3p inhibit oncogene activity and enhance apoptosis, with their loss often associated with adverse outcomes. Among these, miR-155 and miR-21 are particularly well studied, playing central roles in both tumor progression and remodeling of the tumor microenvironment. This review summarizes current evidence on the biological and clinical relevance of miRNAs in DLBCL, emphasizing their diagnostic and prognostic potential.

## 1. Introduction

Diffuse large B-cell lymphoma (DLBCL) is a highly aggressive type of non-Hodgkin lymphoma (NHL), characterized by extensive molecular and pathological heterogeneity. This variation is reflected in its clinical behavior, resulting in diverse therapeutic responses and prognoses [1,2]. DLBCL is the most frequent form of NHL, with incidence rates over recent years. Rituximab (R), a monoclonal antibody targeting the CD20 antigen on B-cell surfaces, plays a central role in the treatment of DLBCL. Its addition to the CHOP regimen—comprising cyclophosphamide, doxorubicin, vincristine, and prednisolone—markedly improved 5-year overall survival (OS) rates (58% compared to 45%). As a result, the R-CHOP combination became the established first-line therapy for DLBCL patients for many years [3]. R-CHOP induces durable remission in 50–70% of patients; however, up to 30% relapse and 20% present with primary refractory disease [4,5,6]. In first-line treatment, substitution of vincristine with polatuzumab vedotin, an antibody–drug conjugate targeting CD79b, resulted in improved progression-free survival (PFS) but did not significantly affect OS [7]. DLBCL may arise through the transformation of a pre-existing indolent lymphoma, such as follicular lymphoma (FL) and marginal zone lymphoma. Nonetheless, the majority of DLBCL cases arise de novo, without evidence of a preceding indolent lymphoma [8].

Molecular analyses using gene expression profiling classify DLBCL into three distinct subtypes: the germinal center B-cell–like (GCB) subtype, the activated B-cell–like (ABC) subtype, and a third category referred to as type 3 or unclassified cases [9]. More recently, advanced taxonomic systems have provided a more detailed classification of DLBCL based on comprehensive molecular and cytogenetic analyses [10]. A proposed molecular framework includes the following genetic subtypes: (a) MCD, characterized by co-mutations in MYD88 L265P and CD79B; (b) BN2, defined by BCL6 fusions or NOTCH2 mutations; (c) N1, associated with NOTCH1 mutations; and (d) EZB, involving EZH2 mutations or BCL2 translocations. Despite these advances, a substantial subset of patients still does not fit into any of these defined categories [11]. A genomic analysis of 304 DLBCL cases led to the proposal of an alternative molecular classification, comprising five distinct clusters (C).

The C1 cluster is distinguished by NOTCH2 mutations and is associated with favorable outcomes, while the C2 cluster is associated with aneuploidy and biallelic inactivation of TP53, resulting in adverse clinical outcomes. C3, defined by BCL2 mutations and translocations, along with mutations in genes involved in epigenetic regulation, is also linked to adverse prognosis; C4 is defined by recurrent alterations in signaling pathways, including JAK/STAT, and is generally associated with more favorable clinical outcomes. In contrast, C5 encompasses tumors characterized by chromosome 18q gains and concurrent MYD88 and CD79B mutations, a molecular profile typically linked to poor prognosis. In addition, this study systematically evaluated the prognostic significance of multiple genetic alterations across these molecular subgroups. Notably, gain of 13q31.3—which includes the miR-17-92 cluster—was identified as an independent predictor of worse progression-free survival (PFS), highlighting the potential role of miRNA dysregulation in the pathogenesis and progression of DLBCL [12]. The recently developed classification systems for DLBCL establish more accurately defined subtypes through integrated genetic, molecular, and immunophenotypic data, in order to better reflect the disease’s complexity and to support a more tailored and effective therapeutic approach. Nonetheless, these classifications rely on advanced molecular methodologies that are not yet routinely available in clinical settings or still require additional validation for widespread clinical use [13,14].

To better stratify DLBCL patients into defined risk groups and more precisely predict clinical outcomes, several prognostic scoring systems have been introduced. The most widely used among them is the International Prognostic Index (IPI), although it was developed prior to the introduction of rituximab. This index considers factors such as patient age, Ann Arbor stage, circulating lactate dehydrogenase (LDH) concentrations, ECOG performance status, and the number of disease sites. Based on these parameters, patients are classified into four risk categories—low, low-intermediate, high-intermediate, and high—with corresponding 3-year OS estimates ranging from 91% in the low-risk group to 59% in the high-risk group [15]. Following the integration of rituximab into standard treatment protocols, the revised International Prognostic Index (R-IPI) was introduced. While it retained the original risk factors, it simplified patient stratification into three prognostic groups: very good (no risk factors), good (one to two risk factors), and poor (three to four risk factors) [16]. In addition, the NCCN-IPI was specifically developed for patients with newly diagnosed DLBCL who are candidates for R-CHOP–based immunochemotherapy [17]. This prognostic model was derived from analyses of large patient cohorts within the National Comprehensive Cancer Network (NCCN) database. The NCCN-IPI retains many of the adverse prognostic factors incorporated in earlier scoring systems, while refining their weighting to improve risk stratification and prognostic accuracy in the contemporary treatment era. However, it introduced more refined risk stratification, categorizing patients once again into four risk groups: low, low-intermediate, high-intermediate, and high. Among the three scoring models, the NCCN-IPI appears to offer the greatest predictive accuracy for both PFS and OS. Nonetheless, despite the availability of multiple prognostic models, none have consistently succeeded in clearly delineating a distinct subgroup of patients with an exceptionally poor prognosis. This limitation underscores a persistent unmet need for more refined and accurate risk stratification strategies in DLBCL [18,19]. A more recent prognostic tool for DLBCL patients, known as the International Metabolic Prognostic Index, has been introduced. This prognostic model integrates key clinical and imaging-based variables, including patient age, disease stage, and baseline metabolic tumor volume (MTV), with MTV being quantified using positron emission tomography (PET) imaging [20].

There is a clear need to improve prognostic evaluation in patients with DLBCL, which may be addressed through the identification and validation of novel biomarkers by multiple research groups. This can be achieved by exploring the use of novel biomarkers from multiple research groups. Among the emerging biomarkers, circulating tumor DNA (ctDNA) has gained considerable attention, especially after early studies in patients with non-hematologic solid malignancies. Circulating tumor DNA (ctDNA) refers to short fragments of tumor-derived DNA, generally spanning from approximately 70 base pairs up to 21 kilobases in length, that are detectable in the peripheral circulation. These DNA fragments are released by malignant cells through multiple biological processes, including programmed cell death (apoptosis), cellular necrosis, and, in some cases, active secretion mechanisms [21]. Numerous studies have demonstrated the promise of ctDNA as a minimally invasive biomarker in DLBCL, with potential applications in prognostic stratification, real-time monitoring of therapeutic response, and the early identification of disease relapse [22]. A systematic review of ctDNA as a liquid biopsy in DLBCL identified IGH rearrangements and somatic mutations as the most promising markers for treatment response, while the prognostic value of ctDNA levels and methylation patterns remains unclear and requires further study [23]. Several clinical trials of emerging therapies, including polatuzumab vedotin and odronextamab, have notably utilized ctDNA to assess molecular response. Studies have shown that patients with undetectable ctDNA levels during treatment or upon its completion generally experience longer survival. Therefore, ctDNA could potentially support clinical decision-making by guiding treatment escalation or de-escalation at specific time points [24].

miRNAs belong to a large class of endogenous, naturally occurring single-stranded RNA molecules, approximately 22 nucleotides in length. They regulate gene expression at the post-transcriptional level by binding to target RNAs in a sequence-specific manner [25,26]. Victor Ambros and Gary Ruvkun were awarded the Nobel Prize in Physiology or Medicine in 2024 for their pioneering discovery and characterization of miRNAs, first identified in the roundworm Caenorhabditis elegans [27,28,29]. Since their discovery, it is estimated that miRNAs regulate nearly two-thirds of all human genes [30]. miRNAs participate in the regulation of numerous biological processes [31], such as cell proliferation, differentiation, development, and programmed cell death (apoptosis) [32]. Their dysregulation has been linked to various human diseases [33], including diabetes, cardiovascular conditions, and cancer [33,34]. Importantly, contemporary molecular and genetic classification systems of diffuse large B-cell lymphoma also highlight the biological relevance of miRNA dysregulation. Several genetically defined subtypes are characterized by alterations in signaling pathways or chromosomal regions that directly involve miRNA loci or miRNA-regulated networks [2,11,12]. Gain of chromosome 13q31.3, encompassing the miR-17-92 cluster, represents a prominent example and has been associated with aggressive disease biology and inferior clinical outcome [12]. Likewise, overexpression of miR-155 and miR-21 is closely linked to activated B-cell–like DLBCL and genetic subtypes driven by chronic B-cell receptor and NF-κB signaling, including MCD and C5 clusters [35,36]. Conversely, tumor suppressor microRNAs such as miR-34a, a direct transcriptional target of TP53, are frequently downregulated in molecular subgroups characterized by TP53 inactivation and genomic instability [37]. Together, these observations indicate that miRNA dysregulation is tightly integrated into the molecular architecture that underpins modern DLBCL taxonomies, acting as a functional bridge between genetic alterations, oncogenic signaling pathways, and clinical heterogeneity. miRNAs are considered promising biomarkers, as they can be detected in both tumor tissues and peripheral blood, and they remain stable during sample processing and storage [38]. This review provides an overview of miRNA biogenesis and focuses on the role of miRNAs in the pathogenesis of DLBCL. Additionally, it discusses their potential utility as biomarkers in patients with DLBCL.

## 2. miRNAs Biogenesis

MiRNA biogenesis begins with the processing of transcripts generated by RNA polymerase II or III, processed either during transcription or shortly after it [39]. Approximately half of all identified miRNAs are located within genes (intragenic), primarily derived from introns and, to a lesser extent, from exons of protein-coding genes. The rest are located between genes (intergenic), transcribed independently from their own promoters and not associated with host genes [40,41]. The primary and most common route for miRNA production is the canonical biogenesis pathway. In this process, primary miRNA transcripts (pri-miRNAs) are first produced from miRNA genes and then cleaved into precursor miRNAs (pre-miRNAs) by the microprocessor complex. This complex is made up of the RNA-binding protein DGCR8 (DiGeorge Syndrome Critical Region 8) and the RNase III enzyme Drosha [42]. DGCR8 identifies specific sequence motifs within the pri-miRNA, such as the N6-methyladenylated GGAC motif [43]. Drosha, then, cuts the pri-miRNA at the base of its typical hairpin structure, generating a precursor miRNA (pre-miRNA) with a two-nucleotide 3′ overhang [44]. After pre-miRNAs are produced, they are transported from the nucleus to the cytoplasm by the exportin 5 (XPO5)/RanGTP complex. Once in the cytoplasm, the RNase III enzyme Dicer processes them further by removing the terminal loop, producing a mature miRNA duplex [42,45]. The strand direction defines the naming of the mature miRNA: the 5p strand comes from the 5′ end of the pre-miRNA hairpin, while the 3p strand comes from the 3′ end [46]. Both strands can be incorporated into Argonaute (AGO) proteins (AGO1–4 in humans) through an ATP-dependent mechanism [47]. The resulting miRNA duplex is then loaded onto an Argonaute protein, a critical step that promotes assembly of the RNA-induced silencing complex (RISC), a ribonucleoprotein complex. RISC primarily consists of Argonaute 2 (Ago2) and the transactivation-responsive RNA-binding protein (TRBP) [48].

**Figure 1:**
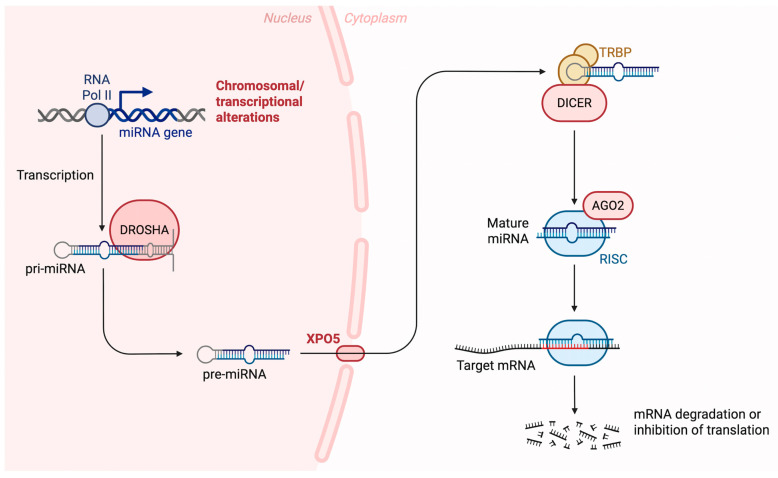
miRNAs are transcribed by RNA polymerase II as primary transcripts (pri-miRNAs), which are then processed in the nucleus by the enzyme Drosha along with its cofactor DGCR8 into precursor miRNAs (pre-miRNAs). These pre-miRNAs are transported to the cytoplasm via exportin 5, where they associate with the Dicer/TRBP complex and are cleaved into short double-stranded RNA molecules. One strand of this miRNA duplex is then incorporated into the Argonaute protein to form the RNA-induced silencing complex (RISC). RISC then binds to specific target mRNAs, leading to their degradation, destabilization, or inhibition of translation. In addition to the canonical pathway, miRNAs can be generated through non-canonical biogenesis routes, including Drosha-independent mechanisms (where pri-miRNAs are processed by the spliceosome) as well as Dicer-independent pathways. Abbreviations: Ago2: Argonaute 2, DGCR8: DiGeorge syndrome critical region 8, RISC: RNA-induced silencing complex, TRBP: Transactivation response element RNA-binding protein. Created in https://BioRender.com (accessed on 8 July 2025). Modified from Seyhan, Attila 2023 [51].

The RISC helps locate target mRNAs by binding to sequences that are complementary, typically within the 3′ untranslated region (3′ UTR) of the mRNA. In humans, perfect base pairing is not required for this interaction to suppress gene expression, which may occur through mRNA degradation, destabilization, or inhibition of translation [49]. The biosynthesis of miRNAs is depicted in Figure 1. Because pri- or pre-miRNAs can be imprecisely cleaved at either the 5′ or 3′ end, a single miRNA gene may give rise to multiple isoforms, known as isomiRs. These different isomiRs can target various genes and signaling pathways, even when originating from the same miRNA. Moreover, certain isomiRs are specific to particular cancer types, highlighting their potential as promising biomarkers for future research and diagnostics [50].

## 3. miRNAs and Their Role in DLBCL Pathogenesis and Prognosis

MiRNAs are key regulators in the development of DLBCL and influence how the disease responds to treatment [52]. Several miRNAs, such as miR-155, miR-21, and the miR-17-92 cluster, act as oncogenes by suppressing tumor-suppressor genes, thereby enhancing cell growth, survival, and the ability to evade the immune system. In contrast, miRNAs like miR-34a, miR-144, and miR-181a serve as tumor suppressors by downregulating oncogenes such as SIRT1, BCL6, and CARD11 [52,53]. Accumulating evidence indicates that non-coding RNAs, including both miRNAs and lncRNAs, regulate key oncogenic and immune-related signaling pathways in lymphoma, such as PI3K/AKT, JAK/STAT, Wnt/β-catenin, and NF-κB, by modulating transcriptional, post-transcriptional, and epigenetic mechanisms [54]. Importantly, several lncRNAs act as molecular sponges or scaffolds for miRNAs, thereby fine-tuning pathway activity and contributing to lymphoma cell survival, immune evasion, and drug resistance [54]. These mechanistic insights provide a strong pharmacological rationale for targeting ncRNA-regulated signaling networks as adjunct or novel therapeutic strategies in lymphoma [54]. Presented below is an overview of each miRNA and its association with the pathogenesis of DLBCL.

### 3.1. miR-155

MiR-155 is a well-characterized oncomiR encoded by the B-cell integration cluster (BIC) gene located on chromosome 21 [55]. Originally identified through its activation following avian leukosis virus insertion in B-cell lymphomas, miR-155 has subsequently emerged as a key regulatory molecule involved in a wide range of biological processes. Extensive research has demonstrated its central role in the modulation of immune responses, regulation of hematopoietic differentiation, and control of inflammatory pathways, underscoring its importance in both normal physiology and disease pathogenesis [56]. MiR-155 has been shown to influence the differentiation of T helper cells and the germinal center (GC) response, helping to generate an effective antibody response dependent on T cells, partly by controlling the production of various cytokines [57]. MiR-155 regulates the development of myeloid cells and the production of inflammatory cytokines by targeting SOCS1 and SHIP1, both of which are negative regulators of the PI3K/Akt signaling pathway. Its abnormal expression has been linked to various hematologic and solid tumors, which is why it is classified as an oncomiR [58].

The cancer-promoting role of miR-155 was demonstrated in Eµ-miR-155 transgenic mice, which developed aggressive pre-B-cell tumors within 3 to 4 weeks. In this model, miR-155 was also shown to promote B-cell malignancies by reducing the expression of SHIP1 and C/EBPβ proteins [59]. Within the IL-6 signaling cascade, SHIP1 and CEBPβ function as key regulatory factors that modulate downstream cellular responses. When miR-155 is upregulated, it suppresses the expression of these genes, leading to impaired B-cell differentiation and enhanced cell survival. This survival advantage is linked to the activation of the PI3K/Akt and MAPK signaling pathways [59,60]. PI3K inhibitors have been evaluated in clinical trials involving patients with relapsed or refractory DLBCL, with emerging evidence suggesting that their therapeutic efficacy may be enhanced in tumors exhibiting high levels of miR-155 expression [61]. Beyond its previously described targets, miR-155 has also been shown to negatively regulate the Human Germinal-center Associated Lymphoma (HGAL) gene. HGAL is selectively expressed in GC B cells and contributes to the regulation of lymphocyte and lymphoma cell motility by activating the RhoA signaling pathway and through direct interactions with cytoskeletal components, including actin and myosin [62,63]. Among patients with DLBCL, the expression of HGAL has been shown to correlate with superior overall survival, regardless of the IPI score [59,62]. When miR-155 is overexpressed, it suppresses HGAL, which may contribute to increased lymphoma cell spread and tumor aggressiveness [63].

miR-155 has also been shown to function as an important regulatory factor in immune responses [64]. Exosomes, which are small lipid-bilayer–enclosed extracellular vesicles, are increasingly recognized as important mediators of intercellular communication [65]. These vesicles can carry miRNAs, such as miR-155, and facilitate their transfer between cells [65,66]. Dendritic cells (DCs) release exosomes containing miR-155, which can influence and modulate the inflammatory response [67]. The anti-tumor role of miR-155 in DC activity has been substantiated by evidence derived from both in vitro [67] and in vivo [68] experimental studies. These studies demonstrate that miR-155–enriched exosomes enhance dendritic cell–mediated cytokine secretion and facilitate increased tumor infiltration by cytotoxic and helper T lymphocytes, while concurrently reducing the abundance of regulatory T cells [67,68]. The spread of colorectal cancer has been associated with miR-155-containing exosomes that facilitate communication between cancer cells and cancer-associated fibroblasts (CAFs) [69].

Cell-free RNAs, such as miRNAs and circRNAs, have emerged as promising biomarkers for precision medicine strategies in DLBCL [70]. Among them, miR-155 stands out as a promising candidate for diagnostic, prognostic, and predictive use in this lymphoma subtype [38]. As a potential biomarker, miR-155 expression has been investigated in both tissue samples and peripheral blood [36].

Research assessing miR-155 as a diagnostic biomarker in both tissue and blood samples has reported variable and sometimes conflicting results [71,72,73]. Several studies have reported elevated miR-155 expression in tumor tissues and the serum of DLBCL patients compared to non-cancerous lymph nodes, normal peripheral B-cells, and the serum/plasma or exosomal vesicles from healthy individuals [74,75,76]. However, other studies have not found any significant differences in miR-155 levels between DLBCL patients and controls [77,78]. Notably, serum miR-155 levels have been shown to correlate positively with its expression in tumor tissue, suggesting its potential as a minimally invasive blood-based biomarker [76]. Interestingly, miR-155 levels in serum may vary depending on the disease phase—fluctuating at diagnosis, during treatment, or in the event of disease progression [79]. When comparing miRNA profiles between patients under treatment and those with progressive disease, a general decline in miRNA levels has been observed [79].

There is general agreement on the diagnostic value of miR-155 in differentiating between the ABC and GCB subtypes of DLBCL. Multiple studies have shown that miR-155 expression, measured in either tissue or serum, is higher in patients with the non-GCB/ABC subtype than in those with the GCB subtype [73,74,75,76,77], likely due to miR-155 targeting the HGAL gene as previously mentioned [63]. Moreover, miR-155 expression varies markedly when comparing primary (de novo) DLBCL with aggressive B-cell lymphomas that arise through transformation [80], with higher levels observed in de novo cases. However, a separate study examining the transformation of FL to DLBCL identified changes in the expression of other miRNAs (including miR-223, 217, 222, 221, and let-7i/7b) [74]. Notably, miR-155 was not among the miRNAs found to be differentially expressed between de novo and transformed DLBCL in that study [74]. As a result, miR-155 does not appear to be a reliable marker for distinguishing FL from DLBCL or for predicting FL transformation into DLBCL [74].

The role of miR-155 as a prognostic biomarker in DLBCL remains a subject of ongoing debate. One study analyzing 90 tissue samples from patients with de novo DLBCL found that lower miR-155 expression was linked to improved 5-year PFS [64]. Similarly, two other studies reported that elevated miR-155 levels were associated with poorer OS [81]. In a cohort of 118 patients, real-time PCR analysis stratified cases by median miR-155 expression into high and low miR-155 expression, revealed significantly shorter survival in those with higher expression [82]. Contrarily, another study found that in patients with the GCB subtype of DLBCL treated with R-CHOP, high miR-155 levels correlated with more favorable outcomes, independent of IPI score [38]. miR-155 has also been investigated in peripheral blood as a potential prognostic indicator. While one study concluded that serum levels of miR-155 lacked prognostic value [74], later research suggested that elevated circulating miR-155 was independently associated with worse prognosis, regardless of IPI classification [78,83]. As with its diagnostic utility, further prospective, randomized studies are needed to clarify the prognostic significance of miR-155 in DLBCL. These discrepancies underscore the need for standardized quantification methods and large prospective validation studies [36]. Overall, miR-155 represents a promising, but currently inconclusive biomarker in DLBCL, with relevance in disease pathogenesis, subtype differentiation, and potentially treatment response prediction.

The clinical relevance of miR-155 as a diagnostic and prognostic biomarker in DLBCL remains incompletely defined, as published studies have yielded inconsistent findings. These inconsistencies are unlikely to be explained exclusively by intrinsic biological heterogeneity and instead largely reflect methodological variability among studies. Key sources of divergence include differences in cohort size and composition (such as subtype distribution between ABC and GCB DLBCL and inclusion of de novo versus transformed cases), treatment regimens (rituximab-naïve versus R-CHOP–treated populations), types of biological specimens analyzed (tumor tissue, circulating serum or plasma, and exosome-derived miRNAs), as well as analytical platforms used for miRNA quantification (RT-qPCR, microarray, or next-generation sequencing) [73,74,75,76,77]. In addition, the lack of normalization approaches—ranging from endogenous controls such as U6 or miR-16 to global mean normalization—further hampers direct comparison across studies [84]. Together, these methodological disparities complicate the interpretation of miR-155 as an independent biomarker and highlight the necessity for standardized protocols and well-designed prospective studies in homogeneous patient cohorts.

### 3.2. miR-21

MiR-21 is one of the most prevalent and evolutionarily conserved miRNAs. It is expressed in nearly all cell types and has key regulatory roles in both normal physiology and pathological conditions [85]. In DLBCL, miR-21 is consistently overexpressed in tumor tissues, serum, and cell lines [86,87,88,89]. It is considered a canonical oncomiR, implicated in promoting tumor progression through suppression of tumor suppressor genes.

Several studies have elucidated the oncogenic role of miR-21 in DLBCL via the PI3K/AKT pathway, primarily by targeting FOXO1 and PTEN. PTEN functions as a tumor suppressor by negatively regulating the PI3K/AKT signaling cascade and is a well-established direct target of miR-21 [90]. FOXO1, a transcription factor, modulates the expression of genes such as p27, p21, FasL, and Bim, all of which contribute to cell cycle arrest and apoptosis [91]. Upon activation, AKT phosphorylates FOXO1, leading to its cytoplasmic translocation and degradation by the proteasome [92]. To address this, Go et al. screened the expression levels of miR-21, FOXO1 and PTEN in a variety of DLBCL cell lines [93]. Immunohistochemical analysis of human DLBCL tissues revealed FOXO1 expression in 80.8% (126 out of 156) of the cases, displaying varying staining intensities and patterns that were associated with miR-21 levels. Specifically, low miR-21 expression was linked to increased nuclear localization of FOXO1, while high miR-21 levels were commonly associated with FOXO1 localization in the cytoplasm. Inhibition of miR-21 led to increased luciferase activity in reporter constructs containing FOXO1-binding sites from the Bim promoter region, indicating that FOXO1 enhances Bim transcription. Supporting this, the use of the transcriptional inhibitor actinomycin D blocked the upregulation of Bim even after miR-21 inhibition [93]. In human DLBCL tissues, Bim expression tended to show an inverse association with miR-21 levels. Specifically, Bim mRNA levels were lower in samples with high miR-21 expression compared to those with low miR-21. Immunohistochemical analysis categorized Bim expression as low (none to mild) or high (moderate to strong) based on staining intensity. Among patients with high miR-21, low Bim expression was more commonly observed. Conversely, high Bim expression appeared more frequently in the low miR-21 group, although this trend did not reach statistical significance [93].

Recent studies have established that miRNAs are critical regulators of tumorigenesis, representing an additional layer of post-transcriptional gene regulation. Their expression profiles are often strongly associated with specific clinical and pathological features of cancer, enabling their use as biomarkers for distinguishing malignant from non-malignant tissues and for predicting patient outcomes [94]. Among them, miR-21 has been implicated in tumor progression by downregulating tumor suppressor genes such as PDCD4, PTEN, and TPM1 [95]. Wang et al. reported that miR-21 expression was significantly elevated in hepatocellular carcinoma tissues compared to adjacent non-tumorous liver tissues [96], while markedly increased levels of miR-21 have also been detected in the cerebrospinal fluid of patients with primary central nervous system lymphoma compared to healthy controls [97]. In the context of DLBCL, miR-21 expression is upregulated both in serum and in tumor tissue [74]. Additionally, another study reported upregulated miR-21 expression in various DLBCL cell lines, including OCI-Ly1, OCI-Ly3, OCI-Ly4, OCI-Ly7, OCI-Ly8, OCI-Ly10, OCI-Ly18, OCI-Ly19, and HBL [89]. Patients with elevated miR-21 expression levels showed poorer OS compared to those with lower expression. Analysis using a Cox proportional hazards model, which accounted for other survival-related factors in DLBCL, identified miR-21 overexpression as an independent prognostic indicator. These findings suggest that miR-21 plays a significant role in the development and progression of DLBCL. Similarly, Mao et al. reported that serum miR-21 serves as a strong and independent predictor of OS in patients with primary central nervous system lymphoma [98]. Go et al. found that elevated miR-21 expression was significantly linked to shorter progression-free and overall survival in DLBCL patients receiving rituximab-based chemotherapy. miR-21 was also identified as an independent prognostic factor in this setting [93]. Similarly, MG Narducci et al. reported that miR-21 was upregulated in cutaneous T-cell lymphoma and could distinguish between patients with poor and favorable prognosis [99]. Although miR-21 is consistently reported as overexpressed in DLBCL, its diagnostic and prognostic significance shows variability across studies. These differences likely reflect heterogeneity in study design rather than true biological inconsistency, including variations in cohort size, treatment regimens, biological material analyzed (tumor tissue versus circulating samples), and miRNA quantification platforms. Furthermore, inconsistent normalization strategies across studies further limit direct comparability [94,95]. Collectively, these factors should be considered when interpreting the clinical utility of miR-21 as a biomarker in DLBCL.

### 3.3. miR-34

The miR-34 family, which consists of miR-34a and the miR-34b/c cluster, targets the tumor suppressor gene p53. Additionally, the loss of miR-34 expression has been associated with resistance to apoptosis induced by p53-activating agents used in chemotherapy [100,101]. Moreover, knockdown of C-MYC was shown to elevate miR-34a expression levels, reduce forkhead box P1 (Foxp1) expression, and induce apoptosis in DLBCL cells [102]. miR-34 also modulates PI3K/AKT pathway which regulates cell survival and growth. In other words, MiR-34s can suppress cancer cell proliferation and induce cell death by inhibiting genes involved in the PI3K/AKT signaling pathway [103].

In chronic lymphocytic leukemia, altered miR-125a-5p and miR-34a-5p expression may predict Richter transformation. Especially, overexpression of miR-125a-5p or low expression of miR -34a-5p can predict almost 50% of Richter Syndrome 0.5 to 5 years before it occurs and their prediction value can guide clinical doctors to select the most appropriate therapeutic strategy considering the potential of transformation of the CLL into a more aggressive disease [104]. In a study in DLBCL cases, investigators found that the co-occurrence of TP53 mutations and miR-34a methylation was associated with a significantly poor prognosis, with a median survival of 9.4 months (*p* < 0.0001), whereas single alterations in TP53 or miR-34a/b/c promoter methylation did not impact survival [105]. The expression level of miR-34a was significantly reduced in Mucosa-Associated Lymphoid Tissue (MALT) lymphomas and DLBCLs compared to normal gastric tissues and peripheral blood mononuclear cells. Furthermore, low levels of miR-34a along with increased FOXP1, p53, and BCL2 coexpression were indicators of poor prognosis [106]. On the other hand, overexpression of miR-34a was associated with better OS and greater sensitivity to doxorubicin in DLBCL [107].

The ability of miR-34s to regulate gene expression and control essential processes such as cell proliferation, apoptosis, and metastasis has also made them promising candidates for targeted cancer therapies, including lymphomas [103]. In conclusion, miR-34a is a key tumor suppressor in DLBCL with well-defined molecular targets and clinical associations. Its loss contributes to disease progression and poor prognosis, while its restoration shows promise in therapeutic strategies. Further studies are warranted to validate its prognostic utility and optimize delivery of miR-34a–based treatments in clinical settings.

### 3.4. miR-17-92

miR-17-92 is a polycistronic miRNA cluster (consisting of miR-17, miR-18a, miR-19a, miR-19b, miR-20a, and miR-92a) that often is overexpressed in particular solid and lymphoid malignancies. The miR-17-92 miRNA cluster targets the tumor suppressor PTEN and the proapoptotic protein Bim by inhibiting their expression [108]. The miR-17-92 cluster is also known as “oncomiR-1” and plays a crucial role in the cell cycle, proliferation, apoptosis, and other essential cellular processes [109]. The transcription factor MYC activates the expression of miR-17-92 by directly binding to its genomic locus [110]. Moreover, the miR-17-92 cluster modulates the expression of E2F1 or both E2F2 and E2F3 (transcription factors that play a crucial role in cell cycle) [109].

Among the molecular subtypes of DLBCL identified through gene-expression profiling, GC-DLBCL is characterized by elevated levels of miR-17-92 and frequent amplification of the 13q31.3 region, where the miR-17-92 locus resides [110,111]. Expression profiling of the miR-17-92 cluster may help distinguish GC-DLBCL from high-grade FL (overexpressed in DLBCL) [112]. The prognostic value of the miR17-92 cluster is under investigation. Overexpression of miR-18a was linked to shorter OS in DLBCL patients treated with R-CHOP [113]. In addition, investigators have found that overexpression of miR-18, miR-19a, and miR-92a was associated with a significant reduction in OS in patients with B-NHL, including patients with DLBCL, while elevated levels of miR-19a and miR-92a were linked to decreased event-free survival (EFS) [114]. Representative methodological differences among studies evaluating the diagnostic and prognostic value of miR-155, miR-21 and the miR-17–92 cluster are summarized in Table 1.

### 3.5. Other miRNAs of Importance in DLBCL

Beyond these miRNAs, a broader network of miRNAs contributes to DLBCL pathobiology through coordinated regulation of B-cell differentiation programs, survival signaling, and treatment response. Rather than acting as isolated regulators, these miRNAs converge on key oncogenic pathways and biological processes that shape disease heterogeneity and clinical behavior. A prominent example of genetic disruption within this regulatory network is miR-142, the only human miRNA gene found to be recurrently mutated in approximately 20% of DLBCL cases. These mutations often occur in the seed sequences of both miR-142-3p and miR-142-5p, which are critical for target recognition and gene regulation [115]. Known and newly identified targets of miR-142 include CFL2, CLIC4, STAU1, TWF1, AKT1S1, CCNB1 (Cyclin B1), LIMA1, and TFRC. These targets are involved in cell cycle regulation, cytoskeletal organization, and iron metabolism, all of which can influence tumor growth and behavior [116]. Functional studies demonstrate that loss of miR-142 activity reshapes the DLBCL proteome toward a tumor-promoting state while impairing immune-related processes such as MHC-I antigen presentation. [116]. Both miR-142-3p and miR-142-5p are highly expressed in DLBCL, but mutations can lead to loss of function and altered regulation of cellular pathways [71,115,117]. These findings position miR-142 as a key node linking genetic alterations to immune evasion and tumor progression in DLBCL.

In parallel, several miRNAs modulate therapeutic response and cell survival pathways. MiR-22 has emerged as a dynamic regulator of therapy response in DLBCL. Its expression and secretion are modulated in response to R-CHOP chemotherapy, with post-treatment upregulation potentially reflecting alterations in tumor biology or treatment efficacy [118]. Mechanistically, miR-22 regulates key signaling pathways, including p53 signaling, by targeting cell cycle regulators such as CDK6 and CDKN1A (p21), thereby influencing both cell cycle arrest and apoptosis [118,119]. In addition, miR-22 modulates the c-MYC/MYCBP axis and the PTEN-AKT pathway, highlighting its broader role in cell survival and proliferation control. These findings suggest that miR-22 may serve as both a biomarker of treatment response and a candidate for targeted therapy, particularly in molecular subtypes of DLBCL characterized by high miR-22 expression [119].

The tumor-suppressive role of miR-181a further illustrates how miRNAs shape subtype-specific signaling dependencies. In DLBCL, miR-181a functions as a tumor suppressor, especially in the ABC subtype. Its expression is reduced in ABC-like cases compared to the GCB subtype, and reintroduction of miR-181a suppresses tumor cell growth and enhances cell death [120]. miR-181a suppresses several key elements of the NF-κB signaling pathway—such as CARD11, IKBα, p105/p50, and C-Rel—resulting in decreased NF-κB activity [121]. This reduction is critical, as NF-κB signaling supports the survival and proliferation of ABC-like DLBCL cells. Restoration of miR-181a attenuates NF-κB activity, suppresses tumor growth, and enhances apoptosis, consistent with clinical observations linking higher miR-181a expression to improved survival outcomes in patients treated with R-CHOP [122]. These data identify miR-181a as a central regulator of NF-κB-driven survival programs in ABC-like DLBCL [53].

Additional tumor-suppressive miRNAs reinforce this regulatory landscape. miR-144, frequently downregulated in DLBCL, directly targets the transcriptional repressor BCL6, a master regulator of germinal center biology and lymphomagenesis [123]. Through binding to the 3′ untranslated region of BCL6 mRNA, miR-144 reduces BCL6 expression, leading to decreased proliferation and invasiveness of DLBCL cells. Although miR-144 is downregulated in DLBCL, its plasma levels could contribute to a biomarker panel for monitoring lymphoma [53]. However, its standalone diagnostic utility in DLBCL remains to be confirmed through further studies. These data underscore miR-144 as a key regulator of BCL6 and a potential target for therapeutic intervention in DLBCL [53,123].

Similarly, miR-124-3p suppresses DLBCL growth by inhibiting the NFATc1/c-MYC signaling axis, leading to reduced proliferation and increased apoptosis in both in vitro and in vivo models [124,125]. Collectively, these miRNAs define regulatory modules that converge on core pathways governing differentiation state, immune signaling, and therapeutic vulnerability in DLBCL. This integrative perspective underscores the importance of considering miRNA networks, rather than individual miRNAs, in understanding disease biology and in developing clinically relevant biomarker and therapeutic strategies.

A summary of miRNAs associated with DLBCL is presented below (Table 2).

### 3.6. Limitations

Interpretation of published miRNA studies in DLBCL is complicated by substantial heterogeneity in cohort size, treatment regimens, sample sources, and analytical platforms. Differences in RNA extraction methods, detection technologies, and normalization strategies further contribute to variability across studies. Consequently, discordant diagnostic or prognostic associations reported for individual miRNAs may reflect methodological differences in addition to true biological heterogeneity. Standardization of study design and analytical pipelines will be essential for the clinical translation of miRNA-based biomarkers [70,117].

## 4. miRNAs and Their Role in Tumor Microenvironment in DLBCL

The tumor microenvironment (TME) is a critical determinant of DLBCL biology and clinical behavior, influencing immune escape, therapy response, and progression. It comprises a complex network of cellular and noncellular components that surround and interact with the tumor [127]. Large-scale gene expression profiling and immune deconvolution studies in DLBCL have demonstrated that variation in immune cell composition within the TME is strongly associated with clinical outcome and response to immunochemotherapy, underscoring the biological relevance of immune–tumor interactions in this disease [127]. Moreover, comprehensive transcriptomic and spatial profiling analyses have identified immune-rich and immune-depleted microenvironmental states that define biologically and clinically distinct DLBCL subsets with divergent survival outcomes and therapeutic responses [128]. In summary, miRNAs are central to DLBCL pathogenesis through their regulation of oncogenic pathways and impact on the tumor microenvironment.

MiR-155 drives tumor progression and shapes the tumor microenvironment [64]. Moreover, in response to danger signals—such as pathogen-associated molecular patterns (PAMPs) or damage-associated molecular patterns (DAMPs) like IFN-β—macrophages and dendritic cells (DCs) exhibit increased expression of miR-155. This upregulation also influences the behavior of myeloid-derived suppressor cells (MDSCs), a group of immature myeloid cells known for their potent immunosuppressive functions [64,129]. Importantly, the accumulation of MDSCs within the tumor microenvironment is commonly linked to poor clinical outcomes [130]. Furthermore, high miR-155 levels are linked to reduced peripheral CD8+ T cells and impaired T-cell receptor signaling. miR-155 also increases PD-L1 expression on lymphoma cells, promoting immune evasion by inhibiting CD8+ T cell function through the PD-1/PD-L1 pathway. Targeting this axis with PD-L1 blockade can restore CD8+ T cell activity and suppress tumor growth, especially in EBV-associated DLBCL [78]. miRNAs can also regulate cytokine production and cell migration within the TME, as seen with miR-155 targeting DEPTOR, affecting pro-inflammatory cytokine expression and DLBCL cell motility [131].

MiR-21 plays a central role in driving disease progression in B-cell lymphoma [132] with experimental studies demonstrating that its overexpression induces a pre-B-cell malignant lymphoid-like phenotype [133]. Clinically, elevated levels of circulating miR-21 in the serum of DLBCL patients correlate with tumor tissue expression, more advanced disease stages, and poorer OS outcomes [89,134]. Dysregulated tumor microenvironment determines cancer cell chemosensitivity [135]. Zheng et al. demonstrated that forced expression of miR-21 induced chemoresistance in B-lymphoma cells, an effect that was even more pronounced when these cells were co-cultured with immune and endothelial cells—key components of the tumor microenvironment. This aligns with earlier studies showing that miR-21 promotes adhesion of myeloma cells to bone marrow stromal cells and contributes to resistance against chemotherapy [136]. Additionally, miR-21 has been shown to activate inflammatory pathways in HER2-positive breast cancer, thereby diminishing the efficacy of neoadjuvant trastuzumab and chemotherapy [137]. While these studies suggest that miR-21 regulates conserved microenvironment-dependent mechanisms of therapy resistance, their direct causal relevance in DLBCL remains incompletely defined and should be considered hypothesis-generating.

Other miRNAs orchestrate complex interactions within the DLBCL TME, influencing immune cell function, tumor progression, and response to therapy. miR-142-5p is upregulated in immunosuppressive tumor-associated macrophages (TAMs) in DLBCL. Inhibiting miR-142-5p can repolarize these macrophages to a more anti-tumor phenotype, enhancing antibody-dependent cellular phagocytosis and improving immunotherapy outcomes. High miR-142-5p and CD206+ TAMs predict poor response to immunochemotherapy [126]. A prognostic model incorporating four circulating miRNAs (miR-21, miR-130b, miR-155, and miR-28) has been associated with elevated levels of myeloid-derived suppressor cells (MDSCs) and Th17 cells within the TME, promoting immunosuppression and leading to poorer clinical outcomes. These miRNAs are involved in modulating oncogenic pathways, such as Ras signaling, as well as shaping the immune cell landscape in DLBCL [138]. These circulating miRNA signatures likely reflect systemic immune dysregulation that is mirrored within the tumor microenvironment, providing a biologically plausible explanation for their prognostic value [128].

Overall, accumulating evidence supports a central role for miRNA-mediated regulation of the tumor microenvironment in DLBCL, influencing immune escape, disease progression, and therapeutic response. At the same time, despite robust correlative and mechanistic data, functional validation of miRNA-mediated immune modulation in DLBCL remains limited, highlighting the need for integrative experimental and clinical studies prior to therapeutic translation [95]. Modulating specific miRNAs in the TME, such as inhibiting miR-155 or miR-142-5p, therefore represents a promising strategy to enhance immune responses and improve the efficacy of immunotherapies in DLBCL, while requiring careful validation to define clinical applicability [78,126,139].

## 5. Conclusions

DLBCL remains a biologically and clinically heterogeneous disease, with outcomes varying widely despite advances in immunochemotherapy. Mounting evidence shows that miRNAs are key regulators of DLBCL initiation, progression, and treatment response. These small non-coding RNAs modulate apoptosis, proliferation, immune evasion, and drug resistance through post-transcriptional regulation of pivotal signaling pathways.

Among them, miR-21 and miR-155 are the most extensively characterized, while miR-181a, miR-124-3p, miR-144, miR-142, and miR-22 also exhibit strong mechanistic and prognostic relevance. These findings suggest that a restricted subset of miRNAs currently approaches minimal translational thresholds, particularly as candidate biomarkers rather than standalone clinical tools. Diagnostic studies have shown that distinct miRNA expression profiles can discriminate between DLBCL subtypes, differentiate malignant from non-malignant lymphoid tissues, and even detect disease through peripheral blood samples. Nevertheless, inconsistencies—such as the variable diagnostic sensitivity and specificity of miR-155—underscore the need for methodological standardization in sample handling, detection platforms, and data interpretation.

From a prognostic and predictive perspective, miRNAs hold promise in refining risk stratification, guiding treatment selection, and monitoring therapeutic response. Elevated miR-21 and miR-155 levels have been associated with treatment resistance or differential sensitivity to agents such as vincristine, highlighting their potential as biomarkers for personalized medicine. Mechanistic studies further reveal that several miRNAs directly regulate core signaling cascades, including PI3K/AKT, NF-κB, c-MYC, and p53, thereby offering both biomarker and therapeutic opportunities.

The expanding understanding of miRNA biology in DLBCL paves the way for innovative therapeutic interventions. Strategies employing antagomiRs, miRNA mimics, and exosome-based delivery systems are under investigation to inhibit oncogenic miRNAs or restore tumor-suppressive ones. However, successful clinical translation will depend on overcoming challenges related to delivery specificity, off-target effects, and the integration of miRNA therapeutics with established treatment modalities. Importantly, the clinical utility of miRNA-based biomarkers will critically depend not only on biological relevance but also on analytical validity, inter-laboratory reproducibility, and compliance with regulatory requirements for clinical-grade assays.

In summary, while miRNAs represent a powerful and versatile class of regulatory molecules in DLBCL, their incorporation into precision-guided clinical algorithms remains conditional on further evidence. Future research should prioritize the validation of robust miRNA panels, the harmonization of analytical methodologies, and the incorporation of miRNA profiling into prospective clinical trials. Such efforts could ultimately enable precision-guided treatment strategies and novel therapeutics that harness the regulatory potential of miRNAs to improve outcomes for patients with DLBCL. Integrative approaches combining miRNA signatures with genomic and proteomic data hold promise for refining DLBCL classification and, ultimately, enabling more personalized therapeutic strategies grounded in reproducible and clinically actionable biomarkers.

## Figures and Tables

**Table 1:** Methodological characteristics of representative studies evaluating miR-155, miR-21 and miR-17-92 in DLBCL. EFS: Event free survival, GC: Germinal Center, IHC: Immunohistochemistry OS: Overall Survival, RT-qPCR: Quantitative reverse transcription polymerase chain reaction.

miRNA	Sample	Cohort Size	Methodology	Correlation	References
miR-155	Tumor tissue	118	RT-qPCR	High miR-155 associated with shorter OS	[82]
miR-155	Tumor tissue	200	RT-qPCR	High miR-155-lymphoma progression	[78]
miR-155	Serum	60	RT-qPCR	No prognostic significance	[74]
miR-21	Timor tissue	156	IHC/ RT-qPCR	High miR-21 associated with worse OS	[93]
miR-17-92	Tumor tissue/serum	36	IHC/ RT-qPCR	High miR 17-92 in GC-DLBCL	[112]
miR-17-92	Tumor tissue	176	RT-qPCR	High miR-18a associated with shorter OS	[113]
miR-17-92	Tumor tissue	71	RT-qPCR	Reduced OS and EFS	[114]

**Table 2:** Key DLBCL-related miRNAs and their primary functions summarized.

miRNAs	Function	Reference
miR-155-5p	Oncogenic; regulates NF-κB pathway, promotes proliferation, useful for diagnosis and classification	[59,60,61]
miR-21-5p	Oncogenic; regulates PI3K/AKT pathway, promotes cell survival, useful for diagnosis	[46,91,96]
miR-17-92	Oncogenic; alters MYC, SHIP expression, promotes tumorigenesis	[53]
miR-34a	Tumor suppressor; targets SIRT1, inhibits tumor growth	[53]
miR-144	Tumor suppressor; targets BCL6, inhibits tumor growth	[53]
miR-181a	Tumor suppressor; targets CARD11, inhibits tumor growth	[53]
miR-124-3p	Tumor suppressor; inhibits NFATc1/cMYC pathway, suppresses proliferation, promotes apoptosis	[92,124]
miR-142-5p	Promotes immunosuppressive macrophage phenotype, associated with poor response to immunochemotherapy	[126]

## Data Availability

No new data were created or analyzed in this study. Data sharing is not applicable to this article.
